# HIF1A acts as target of XiHuang Pill in the treatment of papillary thyroid cancer by regulating dedifferentiation

**DOI:** 10.3389/fchem.2025.1607067

**Published:** 2025-06-09

**Authors:** Xu-Zhe Yu, Wei-Li Zhu, Hua-Chun Qian, Chun-Jiang Sun

**Affiliations:** ^1^ Traditional Chinese Pharmacy, Shengzhou Traditional Chinese Medicine Hospital, Shengzhou, Zhejiang, China; ^2^ Nursing Department, Shengzhou Traditional Chinese Medicine Hospital, Shengzhou, Zhejiang, China; ^3^ Preventive Medicine Department, Shengzhou Traditional Chinese Medicine Hospital, Shengzhou, Zhejiang, China

**Keywords:** papillary thyroid cancer, Xihuang Pill, HIF1A, protein-protein interaction, TCGA

## Abstract

**Background:**

Papillary thyroid carcinoma (PTC) is the most common subtype of thyroid cancer and has shown a rising incidence globally. Despite its generally favorable prognosis, recurrence and therapeutic resistance remain challenges in clinical management. Traditional Chinese Medicine, particularly Xihuang Pill (XHP), has demonstrated promising anticancer potential in various tumors, but its molecular mechanisms in PTC remain unclear. This study aimed to explore the targets of XHP in the treatment of PTC.

**Methods:**

The active ingredients of XHP were first obtained, and ingredients and PTC-related targets were identified, followed by enrichment analysis. Protein-protein interaction (PPI) network was constructed to determine the key ingredients and targets, and then molecular docking was conducted. Key targets’ prognostic role, correlation with differentiation, and immune infiltration level were analyzed based on the TCGA data. *In vitro* experiments were then performed to validate the role of HIF1A. Finally, the association of clinical characteristics with HIF1A was also assessed.

**Results:**

Firstly, 132 common targets were associated with XHP and PTC, enriched in MAPK, PI3K-AKT, and HIF-1 signal pathways. Five hub genes (CCND1, ESR1, AKT, HIF-1A, BCL2) and 2 ingredients were found, with a favorable combination between them. AKT1 and HIF1A were upregulated in PTC, and high expressions of them were related to poor prognosis (all P < 0.05). Further, only HIF1A was upregulated in the advanced stage of PTC and significantly correlated with the dedifferentiation (all P < 0.05). HIF1A upregulation also correlated with the decrease of activated NK cells abundance in PTC (all P < 0.05), while NK cell abundance showed positive correlation with differentiation level (P < 0.05). HIF1A inhibited differentiation of PTC cells, while XHP suppressed PTC progression and promoted differentiation by downregulating HIF1A. Finally, histopathological type and positive lymph node number correlated with HIF1A expression (all P < 0.05).

**Conclusion:**

This study systematically elucidated the potential mechanisms by which XHP exerts anti-PTC effects, highlighting that HIF1A is a promising target of XHP in the treatment of PTC by regulating dedifferentiation. These findings provide a scientific basis for the application of XHP in PTC.

## 1 Introduction

Thyroid cancer (THCA) is a common endocrine cancer (Sung et al., 2021) and papillary thyroid cancer (PTC) is the most common type. The incidence and mortality rates of THCA in women were obviously higher than that of men (Pizzato et al., 2022). It is estimated that about 43,720 new cases of THCA will be diagnosed in the United States in 2023, and the 5-year relative survival rate is about 98.5% (Laura et al., 2024). At present, numerous mRNA have been proven to be involved in the development of THCA (Liu et al., 2021; Wan et al., 2022; Li et al., 2023). As one treatment option, targeted therapy using radiopharmaceuticals was employed for the treatment of THCA (Cabanillas et al., 2019). It is worth noting that radiopharmaceuticals are widely used in cancer theranostics and nuclear medicine (Makarem et al., 2019; Lengacher et al., 2022; Klika et al., 2021; Klika et al., 2022; Makarem et al., 2025). Nevertheless, common treatments – including chemotherapy – may not always give satisfactory outcomes; for example, due to primary and secondary drug resistance (Lu et al., 2022). In addition, aggressive variants of PTC have been increased frequency (Andrés et al., 2020). Hence, it was necessary to identify more targets and explore the potential medicines for the treatment of THCA and improvement of patient’s prognosis.

Notably, many herbs of Chinese traditional medicine have been proven to improve the prognosis of cancers in clinical research (Zhang et al., 2021; Xu et al., 2022; Cao et al., 2021). Xi Huang Pill (XHP) is a famous and classical Chinese herbal formula based on Niu Huang, Ru Xiang and Mo Yao, and has the functions of clearing heat and detoxification (Xu et al., 2023). Besides, XHP was used to treat some cancers in previous clinical studies, such as breast cancer, lung cancer and cervical cancer (Chen et al., 2023). The experiment results demonstrated that XHP could inhibit the development of tumors through promoting cell apoptosis and inhibiting cell proliferation, invasion and angiogenesis (Xu et al., 2023; Su et al., 2018; Cao et al., 2022). Moreover, some studies reported that XHP could improve the prognosis of some cancers through combining with chemotherapy, targeted drugs and surgery (Mao et al., 2019; Zhang et al., 2023). Nevertheless, the mechanism of XHP in the treatment of THCA was not reported.

In this study, we aimed to explore the potential targets of XHP in the treatment of THCA. As the PTC is the most common type and heterogeneity exists between different types of THCA (Yin et al., 2020), this study only focused on the research in PTC. We first performed network pharmacology to determine the potential targets of XHP and PTC. Besides, we tried to explore the interaction between ingredients and targets by molecular docking. We further identified the most important target from aspects of their expression level, prognostic effect, influence on the differentiation and immune microenvironment in PTC. This study explored the potential therapeutic biomarkers of XHP in the treatment of PTC and revealed the regulatory mechanism.

## 2 Materials and methods

### 2.1 Collection of active ingredients of XHP

All ingredients of the main herbs (Niu Huang, Ru Xiang, and Mo Yao) of XHP were obtained from the Traditional Chinese Medicine Systems Pharmacology Database and Analysis Platform (TCMSP) database (https://old.tcmsp-e.com/tcmsp.php). The active ingredients of 3 herbs of XHP (Niu Huang, Ru Xiang and Mo Yao) were screened out with the threshold of oral bioavailability (OB) ≥30, and drug-like property (DL) ≥0.18.

### 2.2 Collection of XHP and PTC related targets and function enrichment analyses on common biomarkers

After we obtained the 2D structures of ingredients from TCMSP, the ingredients-related genes were predicted in Swiss Target Prediction database (http://swisstargetprediction.ch/). Besides, we obtained the PTC-related genes in GeneCard (https://www.genecards.org/) and DisGeNET (https://www.disgenet.org/home/). Venn analysis was then conducted to select the common targets between the disease and ingredients genes. The common biomarkers were imported into STRING (https://cn.string-db.org/) database to construct the protein-protein interaction (PPI) network. Finally, the PPI network was imported into Cytoscape for further analyzing and beautifying the network using Cytoscape apps.

After the gene expression profile was imported into R software (version 4.3.0), the org.Hs.e.g.,.db and clusterProfiler R package were performed for Gene Ontology (GO) and Kyoto Encyclopedia of Genes and Genomes (KEGG) enrichment analyses using enrichGO and enrichKEGG functions. Besides, the results were visualized by the ggplot2 R package. The GO terms included the cellular component (CC), biological process (BP), and molecular function (MF).

### 2.3 Identification of the key biomarkers

The PPI network of common biomarkers was constructed in Cytoscape. Then, the MCODE method in Cytoscape was used to cluster all the biomarkers, and the cluster with the highest score was regarded as the key cluster and selected for further analysis. Regarding the key cluster, cytohubba method was used to determine the hub targets within it. Furthermore, we predicted the miRNA that could bind to hub genes and constructed an mRNA-miRNA-ingredient network. According to the threshold of degree level >1 and the highest closeness level, the core ingredients and targets among the whole network can be determined.

### 2.4 Binding of core ingredients and targets through molecular docking analysis

Next, we verified the combination of core ingredients and targets through molecular docking analysis. We first obtained the 2D structures of core ingredients from TCMSP, and the 3D structures of the target protein were determined in the PDB online platform (https://www.rcsb.org/). Then, these files were imported into Autodock 4 software for dehydration and hydrogenation, and the results were stored in PDBQT format. Subsequently, the molecular docking was conducted using AutoDock Tools (version 1.5.7). The combination energy < -1.5 kcal/mol was regarded as the valid combination, and their binding pattern was visualized using PyMOL (version 2.5).

### 2.5 Importance evaluation of core targets in the development of PTC

Based on the core targets obtained, we then evaluated their role in the PTC using the dataset from the Cancer Genome Atlas (TCGA) database. The TCGA dataset contained 510 patients with PTC and 58 normal samples, as well as providing the RNA-seq expression profile, clinical characteristics data, and survival data. First, we compared the expression differences of core targets between normal and PTC samples. Then we compared the survival difference of patients between high and low expression groups of core targets using Kaplan-Meier analysis and log-rank test. The expression difference of core targets between early and advanced clinical stages was also compared. In addition, we analyzed the correlation of target expression with the thyroid differentiation score (TDS) in patients with PTC. To further evaluate the importance of core targets on the development of PTC, we used XGBoost machine learning algorithm to rank their importance on the PFI status and PFI time of patients.

### 2.6 Potential role of core targets on the immune microenvironment in PTC

We also explored the role of core targets on the immune microenvironment in PTC. The infiltration abundance of 22 immune cells in PTC was first calculated by the CIBERSORT algorithm based on the RNA-seq data of patients. Then, the infiltration difference of 22 immune cells between target high and low expression groups was compared. Further, the correlation between target expression and infiltration abundance of key immune cells was explored. Finally, the correlation between infiltration abundance of key immune cells and TDS was also assessed.

### 2.7 Potential clinical factors correlated with the expression of core targets in PTC

Finally, we explored the potential clinical factors correlated with the expression of core targets in PTC based on the clinical data of patients. The clinical data included age, gender, race, clinical stage, histopathological subtypes of PTC, disease duration, examined number of lymph nodes, positive lymph nodes, personal medical history, tumor depth, tumor length, tumor width, primary neoplasm focus type, and anatomic site. The univariable linear regression analysis was first used to explore the association of these variables with the expression of core target. Then, the multivariable linear regression analysis was performed to explore the independent factors of core target.

### 2.8 Cell culture and treatment

The human normal thyroid cell line Nthy-ori 3-1 (#CL-0817) and the PTC cell lines TPC-1 (#CL-0643) and IHH4 (#CL-0803) were obtained from Wuhan Pricella Biotechnology Co., Ltd. (Hubei, China). All cell lines were cultured in RPMI-1640 medium supplemented with 10% fetal bovine serum (FBS) and 1% penicillin-streptomycin (P/S) and maintained in a humidified incubator at 37°C with 5% CO_2_.

For treatment, TPC-1 cells were exposed to XHP at concentrations of 10 μmol/L, 50 μmol/L, or 100 μmol/L, while control cells received no treatment.

### 2.9 Cell transfection

TPC-1 cells were transfected with the overexpression plasmid oe-HIF1A to investigate the role of HIF1A in PTC. Briefly, 1.5 × 10^5^ cells were seeded in six-well plates and incubated overnight to a confluence of 50%–70%. The oe-HIF1A plasmid and the corresponding negative control (oe-NC) were transfected using Effectene transfection reagent (QIAGEN, Germany) according to the manufacturer’s instructions. The transfection mixture was added dropwise to the cultured cells, which were incubated under standard conditions (37°C, 5% CO_2_) for 24 h. Transfection efficiency was assessed by qRT-PCR and Western blotting to confirm the overexpression of HIF1A at the mRNA and protein levels. Cells were subsequently used for downstream functional assays.

### 2.10 Cell counting kit 8 (CCK-8) assay

Cells were seeded into 96-well plates at a density of 2000 cells/well in 100 μL medium. After treatment, cells were incubated for 12, 24, 48, and 72 h. Next, 10 μL of CCK-8 reagent was added to each well, followed by incubation at 37°C for 3 h. The optical density (OD) was then measured at 450 nm using a microplate reader.

### 2.11 Colony formation assay

Cells (800 cells/well) were seeded into 6-well plates. The culture medium was refreshed every 2–3 days, and cells were allowed to grow for 7–14 days until visible colonies formed. After incubation, colonies were washed twice with PBS, fixed with 75% ethyl alcohol for 15 min, and stained with 0.5% crystal violet for 10 min. The excess stain was gently washed off with PBS and distilled water, and the plates were air-dried. Colonies were photographed.

### 2.12 Wound healing analysis

TPC-1 cells (5 × 10^5^) were seeded into 6-well plates and cultured to reach approximately 90% confluence. A linear scratch was then made across the cell monolayer using a 200-μL pipette tip. Detached cells were removed by washing twice with PBS, and the medium was replaced with serum-free RPMI-1640. Images of the wound area were captured at 0 h and 24 h post-scratch using an inverted microscope.

### 2.13 Transwell analysis for invasion

Transwell chambers were pre-coated with Matrigel and incubated at 37°C for 30–60 min to solidify. A total of 2 × 10^4^ cells suspended in 200 μL of serum-free RPMI-1640 were seeded into the upper chamber, while 600 μL of RPMI-1640 medium supplemented with 10% FBS was added to the lower chamber. After 24 h of incubation, non-invading cells on the upper surface of the membrane were carefully removed using a cotton swab. Invaded cells on the lower membrane were fixed with 75% ethyl alcohol for 30 min, stained with 0.5% crystal violet for 20 min, and rinsed with PBS. Invaded cells were counted under a light microscope.

### 2.14 Quantitative real-time PCR (qRT-PCR)

Total RNA was extracted from TPC-1 cells using RNeasy Plus Mini kit (QIAGEN). RNA purity and concentration were assessed using a Nanodrop 3,000 spectrophotometer (Hangzhou Allsheng Instruments Co., Ltd., Zhejiang, China). Subsequently, 1 μg of total RNA was reverse-transcribed into complementary DNA (cDNA) using the QuantiTect Reverse Transcription kit (QIAGEN). qRT-PCR was performed using Heiff UNICON Universal Blue qPCR SYBR Green Master Mix (Yisheng Biotechnology Co., Ltd., Shanghai, China) on a LightCycler 480 System (Roche, Switzerland) to detect the expression levels of HIF1A, CD97, NIS, TG, and TPO. The qPCR cycling conditions were as follows: 95°C for 30 s, followed by 40 cycles of 95°C for 3 s and 60°C for 20 s. A melting curve analysis was conducted to verify amplification specificity. Gene expression levels were normalized to GAPDH, and relative expression was calculated using the 2^−ΔΔCt^ method.

### 2.15 Western blotting

Total protein was extracted using RIPA lysis buffer (Beyotime, Shanghai, China). Cell lysates were incubated on ice for 30 min and then centrifuged at 12,000 rpm for 10 min at 4°C to collect the supernatant. Protein concentration was determined using the BCA Protein Assay Kit (Beyotime). Equal amounts of protein were separated via SDS-PAGE and transferred onto PVDF membranes (Yisheng). The membranes were blocked with 5% non-fat milk in TBST for 1 h at room temperature, followed by incubation overnight at 4°C with primary antibodies against HIF1A (#ER 1802-41, Huabio, Hangzhou, China), CD97 (#HA722563, Huabio), NIS (#24324-1-AP, Proteintech, IL, United States), TG (#21714-1-AP, Proteintech), TPO (#ab109383, Abcam, United Kingdom), and GAPDH (#10494-1-AP, Proteintech). After washing with TBST, membranes were incubated with HRP-conjugated secondary antibodies (#SA00010-2, Proteintech) at room temperature for 1 h. Protein bands were visualized using an ECL chemiluminescence reagent (Thermo Fisher Scientific, United States).

### 2.16 Statistical analysis

Categorical data were expressed as n (%) and their differences between 2 groups were compared using the χ^2^ test. Non-normally distributed continuous data were expressed as median (P25, P75) and their differences between 2 groups were compared using the Mann-Whitney U test. Machine learning algorithms were used to rank the importance of variables that differed between the groups. The correlation between 2 continuous data was explored by Spearman method. Experimental results were analyzed using GraphPad Prism 10.1.3 and expressed as mean ± standard deviation from three independent experiments. Comparison between the two groups was conducted using t-test, and those among three or more groups were performed using one-way ANOVA followed by Tukey’s test. P less than 0.05 was considered statistically significant.

## 3 Results

### 3.1 The potential targets of XHP and PTC and function enrichment analysis

**FIGURE 1:**
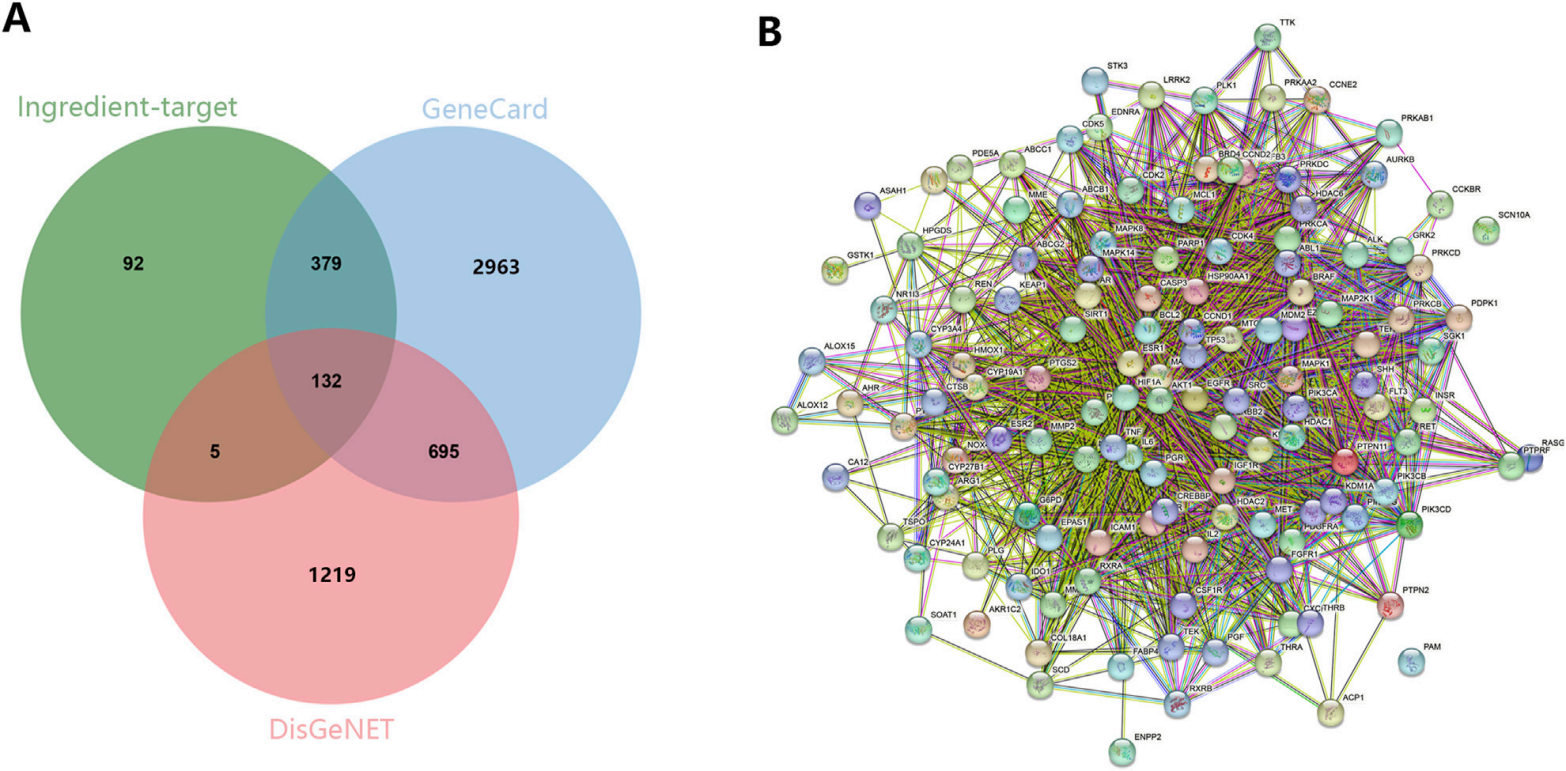
The identification of potential targets. **(A)** The common targets between ingredients and PTC related biomarkers were screened out through the Venn plot. **(B)** PPI network exhibited the interaction among hub genes.

At first, we determined the ingredient of the main herb of XHP through TCMSP by oral bioavailability (OB) ≥30 and drug-like property (DL) ≥0.18. We found that Niu Huang (Bovis Calculus) had 5 active ingredients, Ru Xiang (Olibanum) had 8 active ingredients, and Mo Yao (Myrrha) had 45 active ingredients. Subsequently, we determined the target genes of these active ingredients according to the 2D structure in Swiss Target Prediction. After removing the duplicated targets, a total of 608 targets were obtained. Besides, we obtained PTC related targets through GeneCard (N = 4,169) and DisGeNET (N = 2051). Finally, we got 132 common genes related to disease and ingredients using the Venn plot (Figure 1A). Additionally, we constructed a PPI network of 132 genes through STRING (Figure 1B).

**FIGURE 2:**
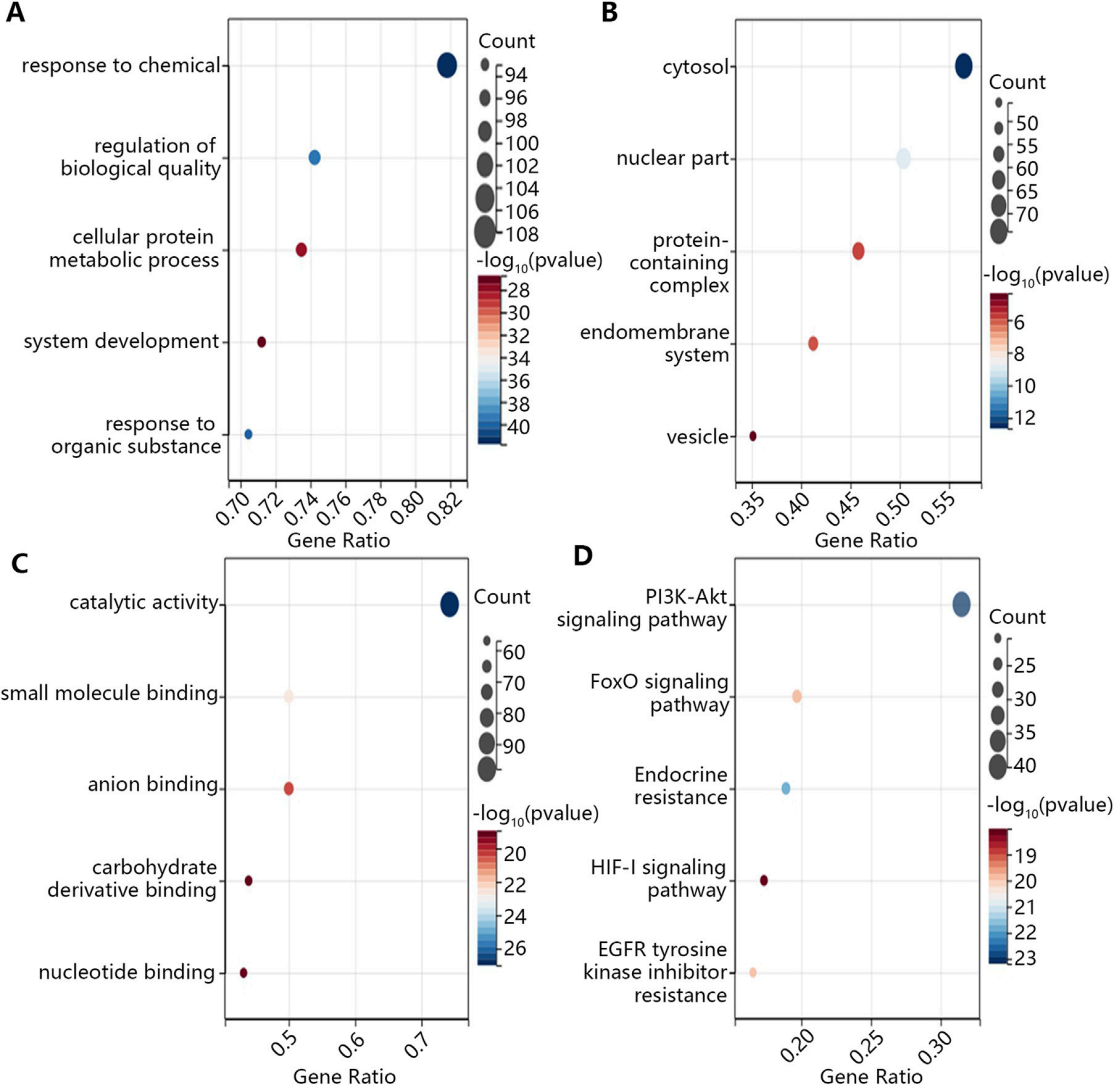
The GO and KEGG enrichment analysis on the common targeted genes. **(A)** BP. **(B)** CC. **(C)** MF. **(D)** KEGG.

Then, we explored the potential function of 132 targeted genes. As shown in Figure 2A, the biological process of these targeted genes was mainly involved in response to chemical, regulation of biological quality and cellular protein metabolic process in the aspect of BP (Figure 2A). Additionally, these genes mainly located in the cytosol, nuclear part and protein-containing complex (Figure 2B). In term of MF, these genes were enriched in catalytic activity, small molecule binding and anion binding (Figure 2C). The results of KEGG enrichment analysis exhibited that targeted genes played vital roles in PI3K-AKT signaling pathway, FoxO signaling pathway and Endocrine resistance (Figure 2D).

### 3.2 Construction of regulatory network and identification of hub genes

**FIGURE 3:**
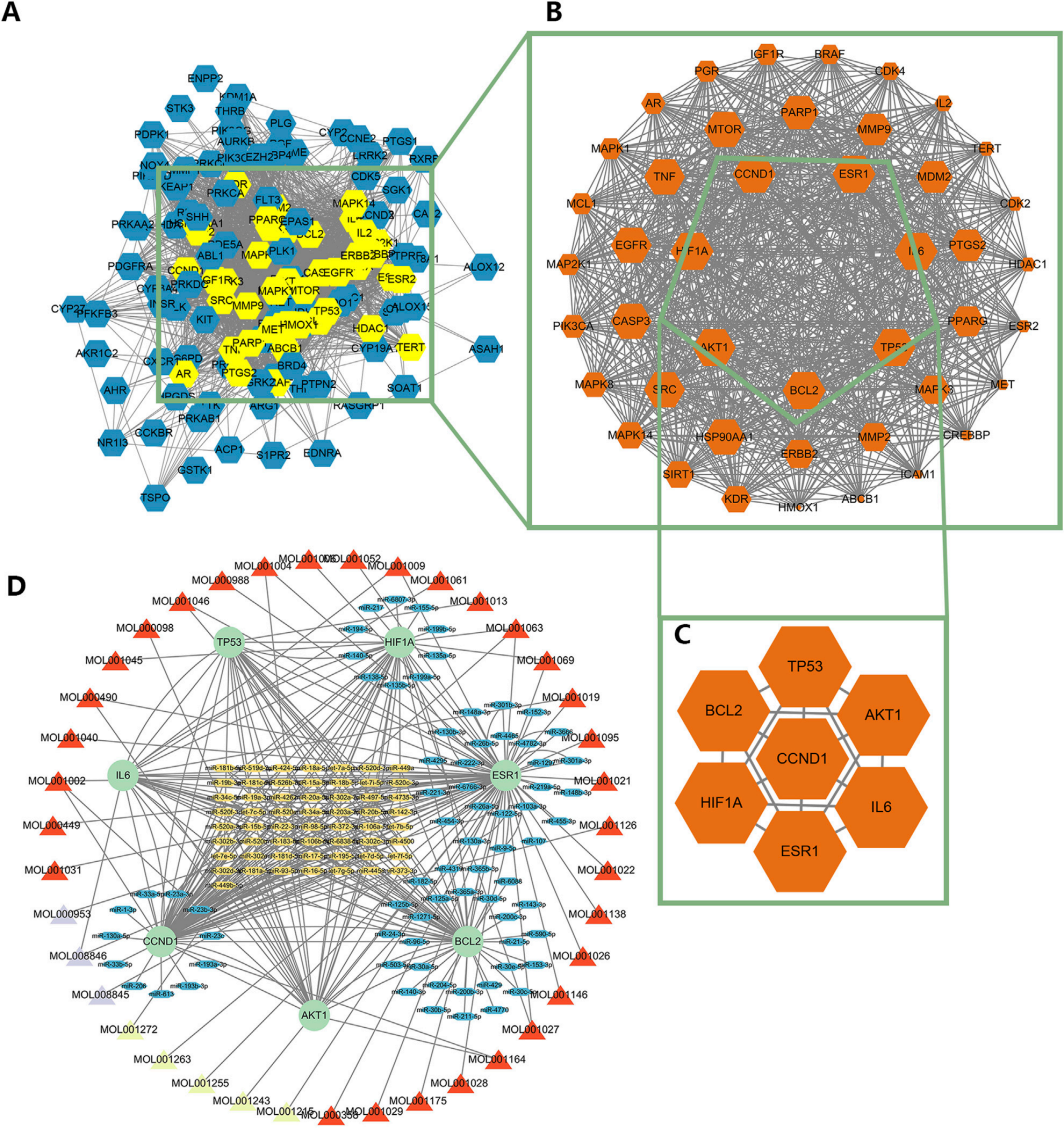
The identification of the main cluster and hub genes and the construction of the regulatory network. **(A)** The interaction among targeted genes, **(B)** The main cluster was identified by MCODE, **(C)** The hub genes with the highest degree level in the main cluster, **(D)** The regulatory network of hub genes. The red triangle meant the ingredient of Mo Yao; The cyan triangle meant the ingredient of Ru Xiang; The lavender triangle meant the ingredient of Niu Huang. The green circle meant the hub genes; The blue oval meant the miRNA only targeted a hub gene; The yellow oval meant the miRNA targeted more than one hub gene.

After the commonly targeted genes were imported into Cytoscape, the main cluster was chosen for further study using MCODE method (Figures 3A,B). Next, 7 hub genes, namely, CCND1, TP53, AKT1, IL6, ESR1, HIF1A and BCL2, were screened out with the highest degree level (Figure 3C). Furthermore, we predicted the miRNA which could bind to 7 hub genes and constructed the mRNA-miRNA-ingredient network (Figure 3D). According to the whole network, the 5 key ingredients with a degree level >1 and highest closeness level were screened out (Supplementary Table S1), including MOL001002, MOL001063, MOL001027, MOL001046, and MOL001069.

These 5 key ingredients all belonged to the main herb of XHP, namely, Mo Yao. The detailed information about the 5 ingredients and 5 core genes connected with ingredients was presented in Tables 1, 2. It should be noted that the 5 core genes all belonged to the 7 hub genes.

**TABLE 1:** The key 5 active ingredients and targets in the whole network.

Mol ID	Molecule name	MW	OB (%)	DL	Connected targets
MOL001002	ellagic acid	302.2	43.06	0.43	CCND1, AKT1, ESR1
MOL001063	28-acetoxy-15α-hydroxymansumbinone	402.63	41.85	0.31	ESR1, CCND1, HIF1A
MOL001027	myrrhanone A	456.83	40.25	1.08	HIF1A, ESR1
MOL001046	(13E,17E,21E)-polypodo-13,17,21-triene-3,18-diol	444.82	39.96	1.05	ESR1, BCL2
MOL001069	3β-acetoxy-16β,20(R)-dihydroxydammar-24-ene	500.89	38.72	0.99	ESR1, BCL2

**TABLE 2:** The detailed information of 5 core targets.

Gene symbol	Aliases	Summary	Genomic locations	Molecular mass
CCND1	Cyclin D1	Protein Coding gene	chr11:69,641,156–69,654,474	33,729 Da
AKT1	AKT Serine/Threonine Kinase 1	Protein Coding gene	chr14:104,769,349–104,795,759	55,686 Da
ESR1	Estrogen Receptor 1	Protein Coding gene	chr6:151,656,672–152,129,619	66,216 Da
HIF1A	Hypoxia Inducible Factor 1 Subunit Alpha	Protein Coding gene	chr14:61,695,513–61,748,259	92,670 Da
BCL2	BCL2 Apoptosis Regulator	Protein Coding gene	chr18:63,123,346–63,320,128	26,266 Da

### 3.3 The combination of 5 active ingredients with targets

**FIGURE 4:**
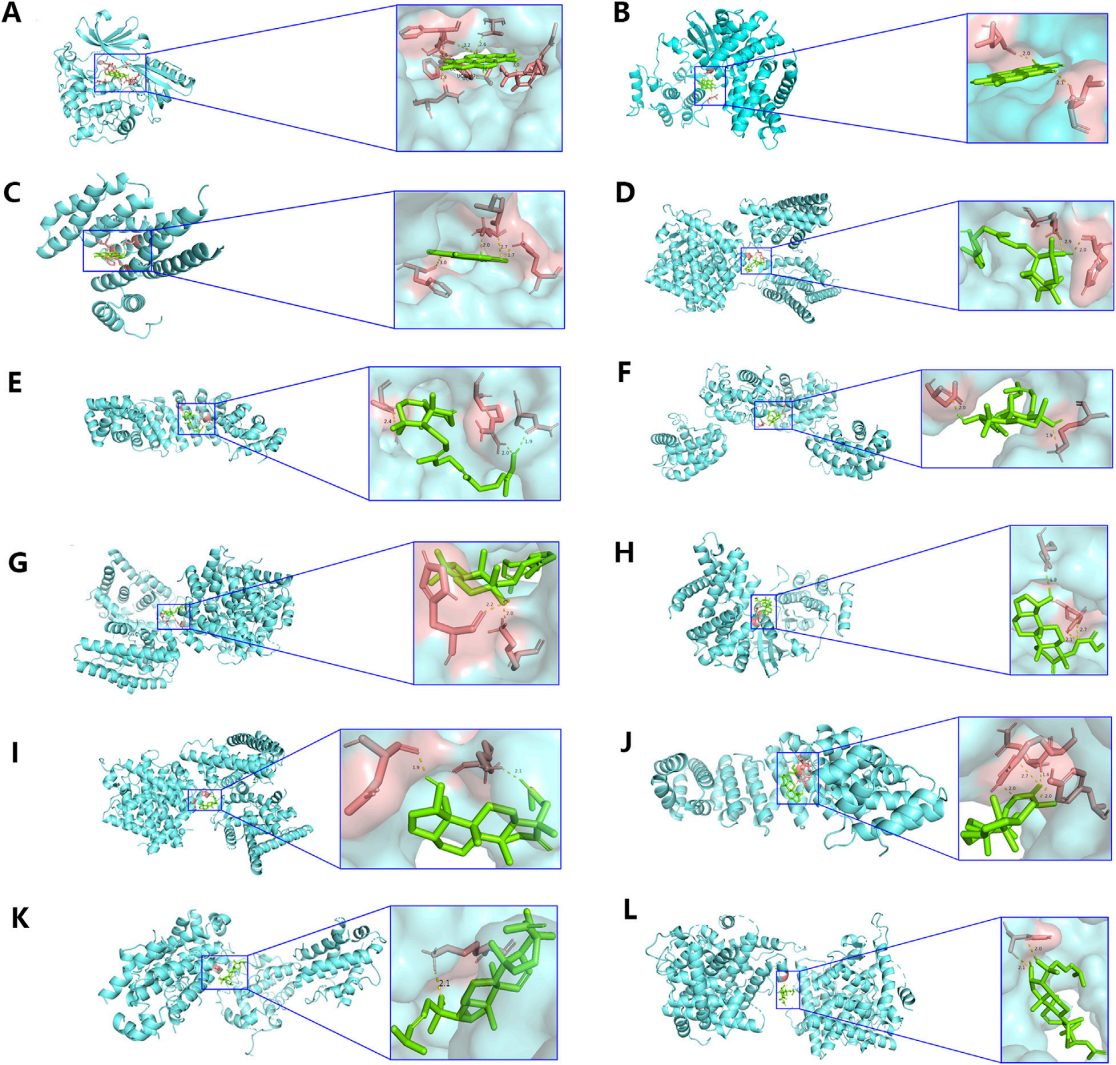
Analysis of molecular docking. The optimal binding conformation between **(A)** MOL001002 and AKT1, **(B)** MOL001002 and CCND1, **(C)** MOL001002 and ESR1, **(D)** MOL001027 and ESR1, **(E)** MOL001027 and HIF1A, **(F)** MOL001046 and BCL2, **(G)** MOL001046 and ESR1, **(H)** MOL001063 and CCND1, **(I)** MOL001063 and ESR1, **(J)** MOL001063 and HIF1A, **(K)** MOL001069 and BCL2, **(L)** MOL001069 and ESR1.

Additionally, molecular docking was performed to explore the binding of 5 molecular drugs with 5 targets using Autodock Vina software. The molecular docking score suggested that they have excellent binding activity (Table 3; Figures 4A–L). These results suggested that the 5 genes may be the potential biomarkers of molecular drugs in the treatment of PTC.

**TABLE 3:** Molecular docking results of the target proteins and active compounds.

Targets	Mol ID	Herb	Docking score (kcal/mol)
CCND1	MOL001002	Mo Yao	−1.52
AKT1	MOL001002	Mo Yao	−6.23
ESR1	MOL001002	Mo Yao	−4.97
ESR1	MOL001063	Mo Yao	−4.99
CCND1	MOL001063	Mo Yao	−3.81
HIF1A	MOL001063	Mo Yao	−4.23
HIF1A	MOL001027	Mo Yao	−5.62
ESR1	MOL001027	Mo Yao	−6.46
ESR1	MOL001046	Mo Yao	−6.27
BCL2	MOL001046	Mo Yao	−5.60
ESR1	MOL001069	Mo Yao	−4.25
BCL2	MOL001069	Mo Yao	−4.09

### 3.4 Importance of HIF1A in PTC development and immune microenvironment

**FIGURE 5:**
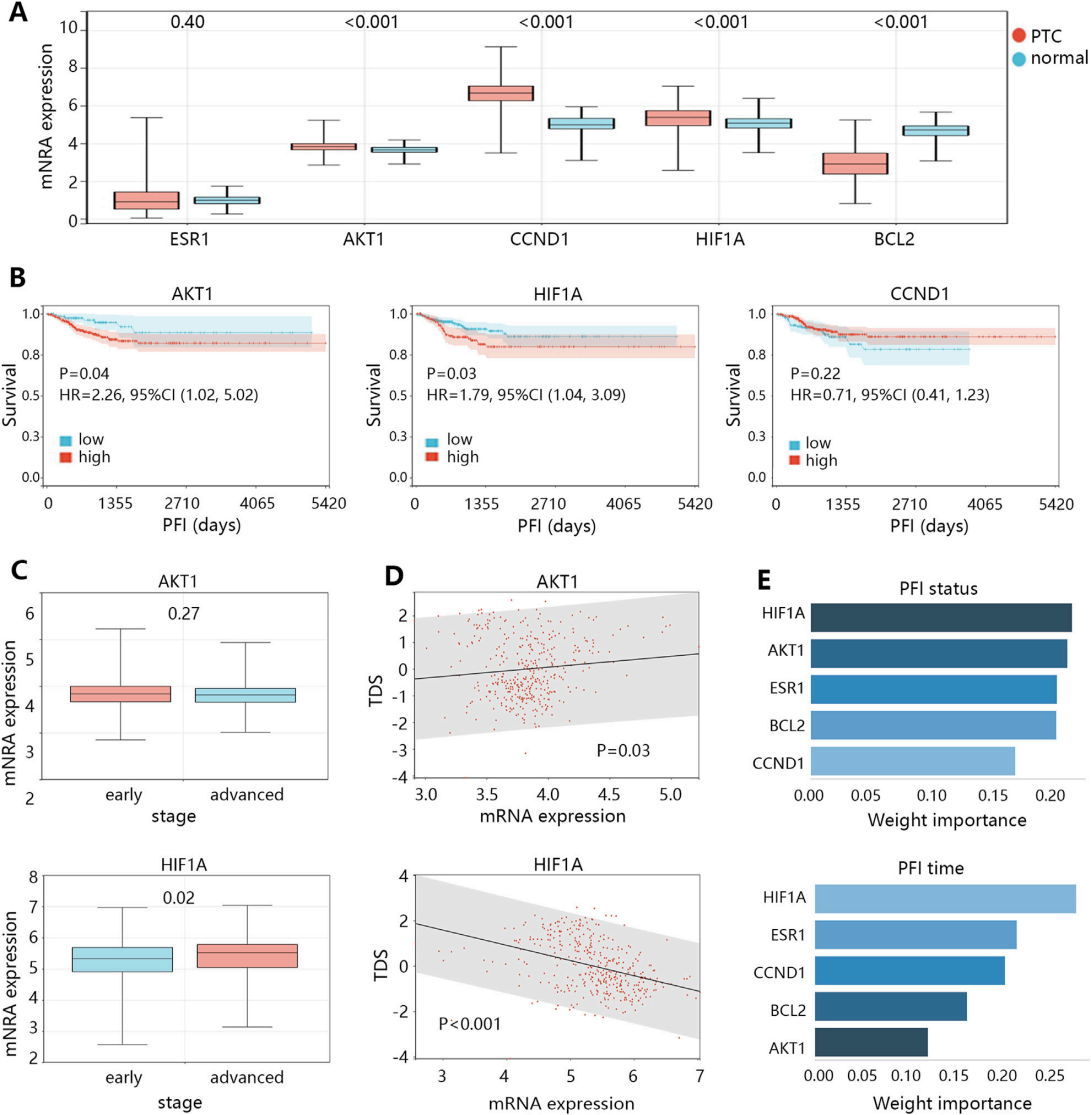
The clinical value exploration of 5 targets in PTC development. **(A)** The mRNA expression differences of 5 targets between normal and PTC groups. **(B)** The survival differences between high and low expression groups of 3 targets in patients with PTC. **(C)** The expression differences of 2 targets between the early and advanced stages of patients with PTC. **(D)** The correlation between expression of 2 targets and thyroid differentiation score (TDS) in patients. **(E)** The importance ranking of targets on the PFI status and PFI time of patients by XGBoost machine learning algorithm.

The above analyses have suggested the 5 genes as potential biomarkers in the treatment of TPC. Next, we explored their role in cancer development. The expression analysis (Figure 5A) showed that ESR1 had no expression difference between normal and tumor groups; BCL2 expression was lower in tumor group (P < 0.001); while AKT1, CCND1, and HIF1A expressions were higher in tumor group compared with those in normal group (all P < 0.001). Expression analysis initially suggested the promotion effect of AKT1, CCND1, and HIF1A in the PTC initiation. We further explored the influence of AKT1, CCND1, and HIF1A on the survival of patients (Figure 5B), finding that high expressions of AKT1 (HR = 2.26, 95%CI: 1.02–5.02, P = 0.04) and HIF1A (HR = 1.79, 95%CI: 1.04–3.09, P = 0.03) were associated with the poor PFI of patients. Survival analysis indicated the role of AKT1 and HIF1A in the progression of PTC. Expression and survival analyses suggested that AKT1 and HIF1A may be the oncogenes in the development of THCA.

It should be noted that the progression of THCA was largely related to the dedifferentiation of cancer cells. Therefore, we subsequently compared the expression differences of AKT1 and HIF1A between early and advanced clinical stage groups (Figure 5C), finding that only HIF1A expression was higher in the advanced stage group (P = 0.02). We also assessed the correlation of AKT1 and HIF1A expressions with the TDS in patients with PTC (Figure 5D), suggesting that only HIF1A showed significant correlation with TDS in a negative pattern (r = −0.38, P < 0.05). In addition, we used XGBoost machine learning algorithm to rank the importance of the 5 targets on the prognosis (PFI status or PFI time), and the results showed that the importance of HIF1A ranked first (Figure 5E). These results highlighted that HIF1A may be the most important biomarker in PTC among 5 targets, and it can cause the PTC dedifferentiation, thus leading to disease progression and poor prognosis.

AKT1, as one of the oncogenes, showed no difference between early and advanced clinical stage groups, and also did not correlate with TDS. The related causes of disease deterioration caused by AKT need to be further explored.

**FIGURE 6:**
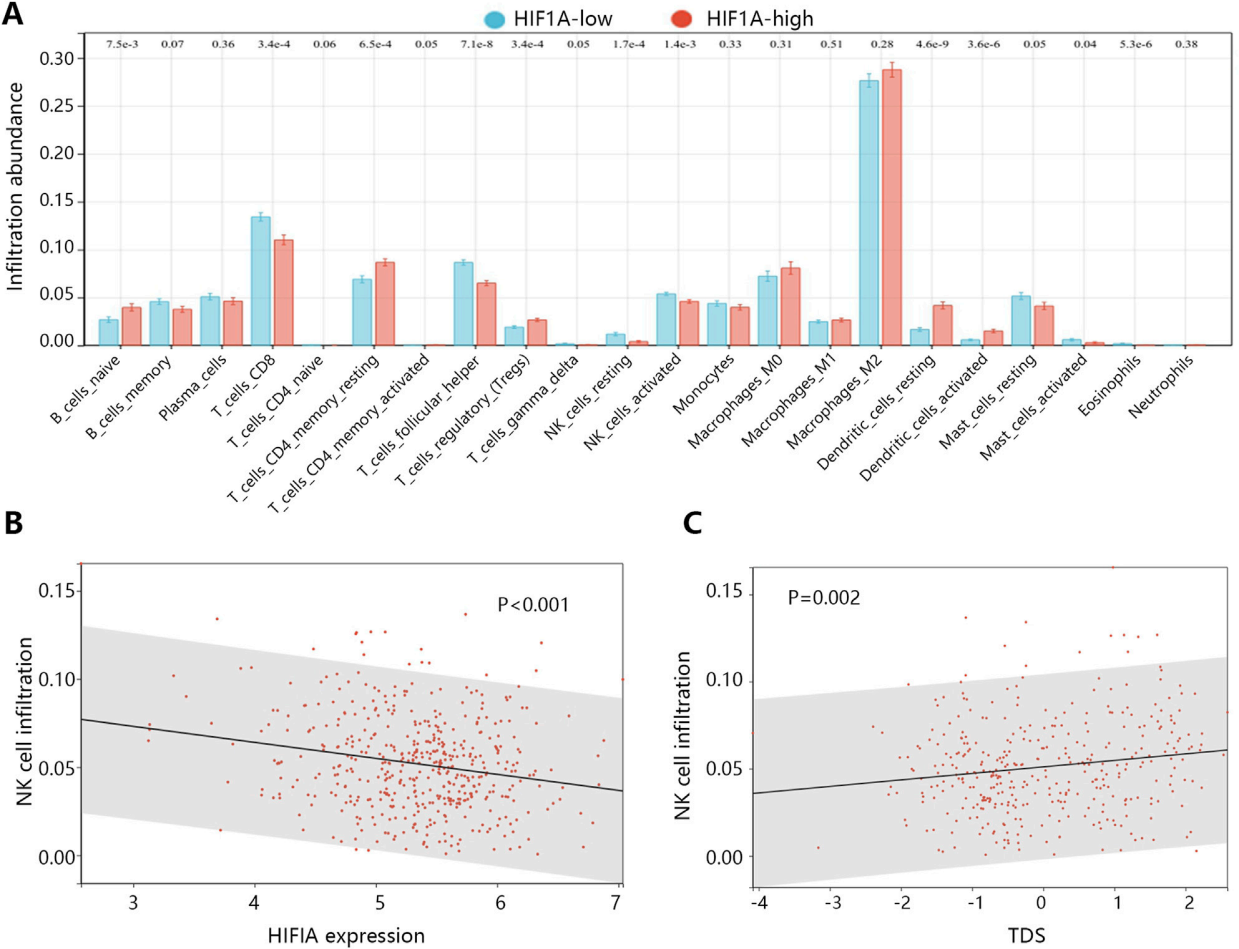
The association of HIF1A with the immune microenvironment in PTC. **(A)** The infiltration abundance differences of immune cells between HIF1A high and low expression groups. **(B)** The correlation between HIF1A expression and infiltration abundance of activated NK cells. **(C)** The correlation between TDS and infiltration abundance of activated NK cells.

In the process of tumor deterioration, besides the changes of tumor cells themselves, the changes of immune microenvironment are also an essential aspect. Therefore, we further explored the association between HIF1A expression and infiltration abundance of immune cells in patients with PTC. The analysis showed that the activated natural killer (NK) cell abundance was significantly decreased in the HIF1A high expression group (Figure 6A, P = 0.001). Correlation analysis further found their significant negative correlation (Figure 6B, P < 0.001), as the activated NK cell abundance decreased with the increase of HIF1A expression. While TDS positively correlated with the NK cell abundance (Figure 6C, P = 0.002).

### 3.5 HIF1A inhibits differentiation of PTC cells

Bioinformatics analysis indicated that HIF1A is closely related to the dedifferentiation of PTC. To validate this finding, we conducted cellular experiments using a normal thyroid cell line (Nthy-ori 3-1) and two PTC cell lines (TPC-1 and IHH4) to examine HIF1A expression. As shown in Supplementary Figure S1A,B, compared to Nthy-ori 3-1 cells, both TPC-1 and IHH4 cells exhibited significantly upregulated HIF1A mRNA and protein expression (P < 0.001). The upregulation of HIF1A was more pronounced in TPC-1 cells than in IHH4 cells. Therefore, all subsequent experiments were conducted in TPC-1 cells.

**FIGURE 7:**
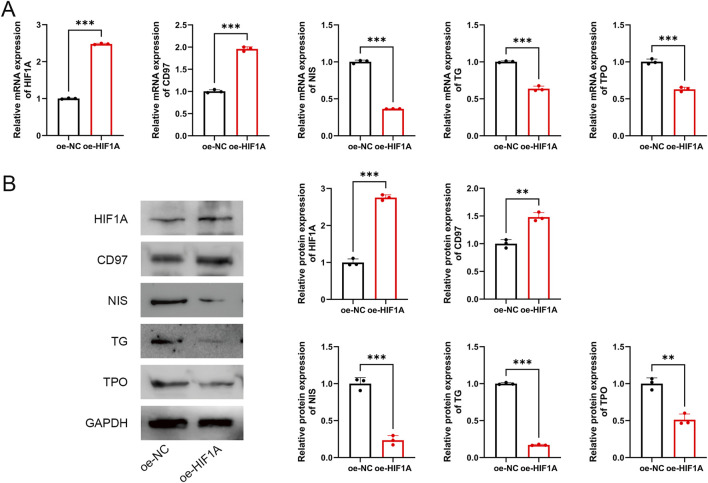
HIF1A inhibits differentiation of PTC cells. **(A)** mRNA expression of HIF1A, CD97, NIS, TG, and TPO. **(B)** Protein expression of HIF1A, CD97, NIS, TG, and TPO. **P < 0.01, ***P < 0.001.

To further investigate the role of HIF1A, TPC-1 cells were transfected with oe-HIF1A to overexpress HIF1A. The results demonstrated that, compared to the oe-NC group, HIF1A expression was significantly upregulated in transfected cells (P < 0.001, Figures 7A,B), confirming successful transfection. Moreover, HIF1A overexpression promoted the expression of the dedifferentiation-related marker CD97 while inhibiting the expression of differentiation-related markers NIS, TG, and TPO (P < 0.01, Figures 7A,B).

### 3.6 XHP inhibits proliferation, migration, and invasion while promoting differentiation of TPC-1 cells

**FIGURE 8:**
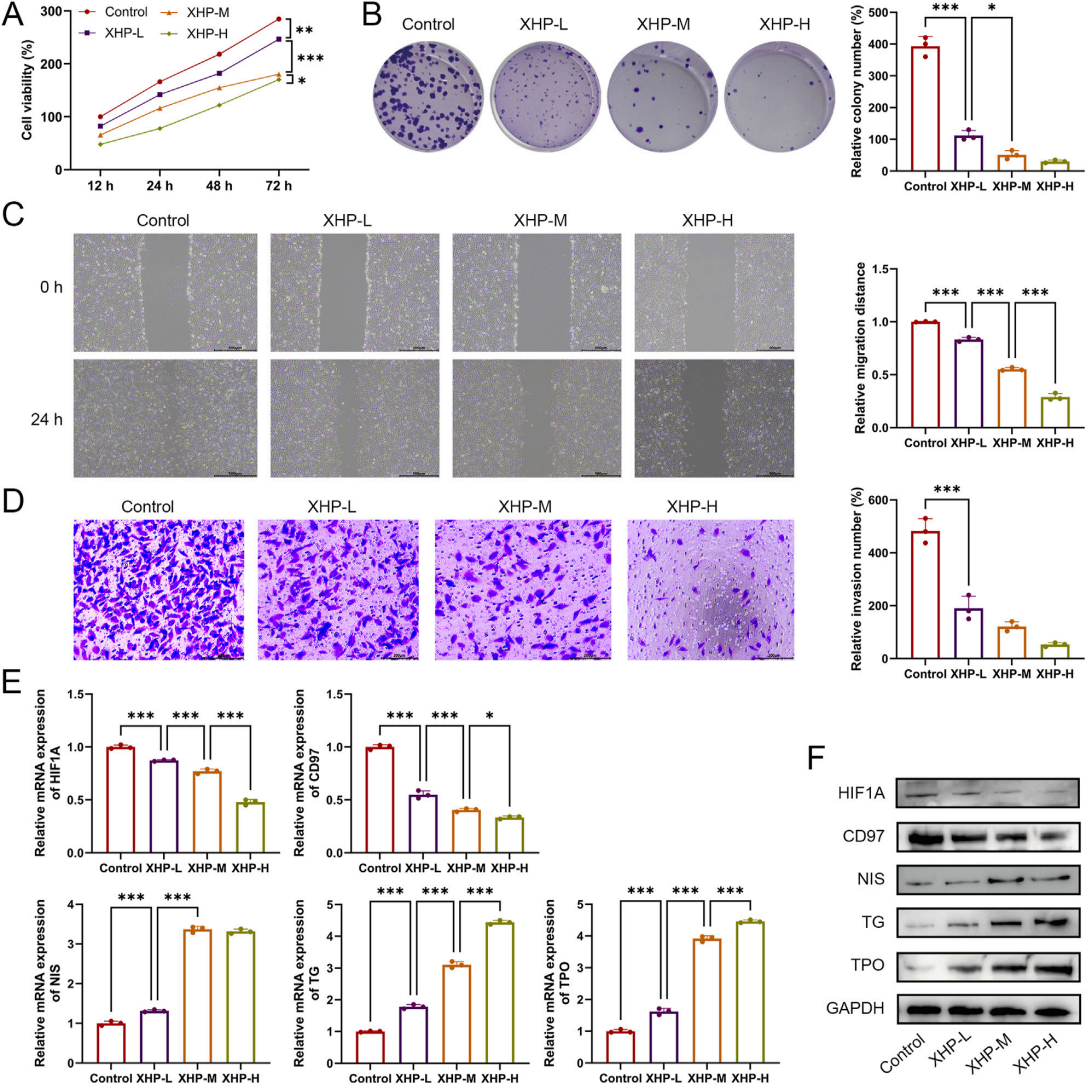
XHP inhibits proliferation, migration, and invasion while promoting differentiation of TPC-1 cells. **(A)** Cell viability was detected using CCK-8. **(B)** Cell proliferation was determined using colony formation analysis. **(C)** Cell migration was determined using wound healing analysis; scale bar = 500 μm. **(D)** Cell invasion was determined using Transwell analysis; scale bar = 200 μm. **(E)** mRNA expression of HIF1A, CD97, NIS, TG, and TPO. **(F)** Protein expression of HIF1A, CD97, NIS, TG, and TPO. TPC-1 cells were treated with 10 μmol/L (XHP-L), 50 μmol/L (XHP-M), or 100 μmol/L (XHP-H) of XHP. *P < 0.05, **P < 0.01, ***P < 0.001.

Next, the therapeutic effects of XHP in PTC were evaluated. TPC-1 cells were treated with 10 μmol/L, 50 μmol/L, or 100 μmol/L of XHP. As shown in Figure 8A, XHP inhibited TPC-1 cell viability in a dose-dependent manner (P < 0.05). Additionally, XHP treatment significantly suppressed TPC-1 cell proliferation, migration, and invasion (P < 0.001, Figures 8B–D). Among the three tested concentrations, the highest concentration of XHP showed the strongest therapeutic effect. Furthermore, compared to the control group, XHP downregulated HIF1A mRNA and protein expression in a dose-dependent manner (Figures 8E,F). XHP also promoted the differentiation of TPC-1 cells, as evidenced by the downregulation of CD97 expression and significant upregulation of NIS, TG, and TPO expression in XHP-treated cells (Figures 8E,F). The effect of XHP on TPC-1 cell differentiation also exhibited a dose-dependent pattern.

### 3.7 XHP inhibits TPC-1 cell malignant features and promotes differentiation by downregulating HIF1A expression

**FIGURE 9:**
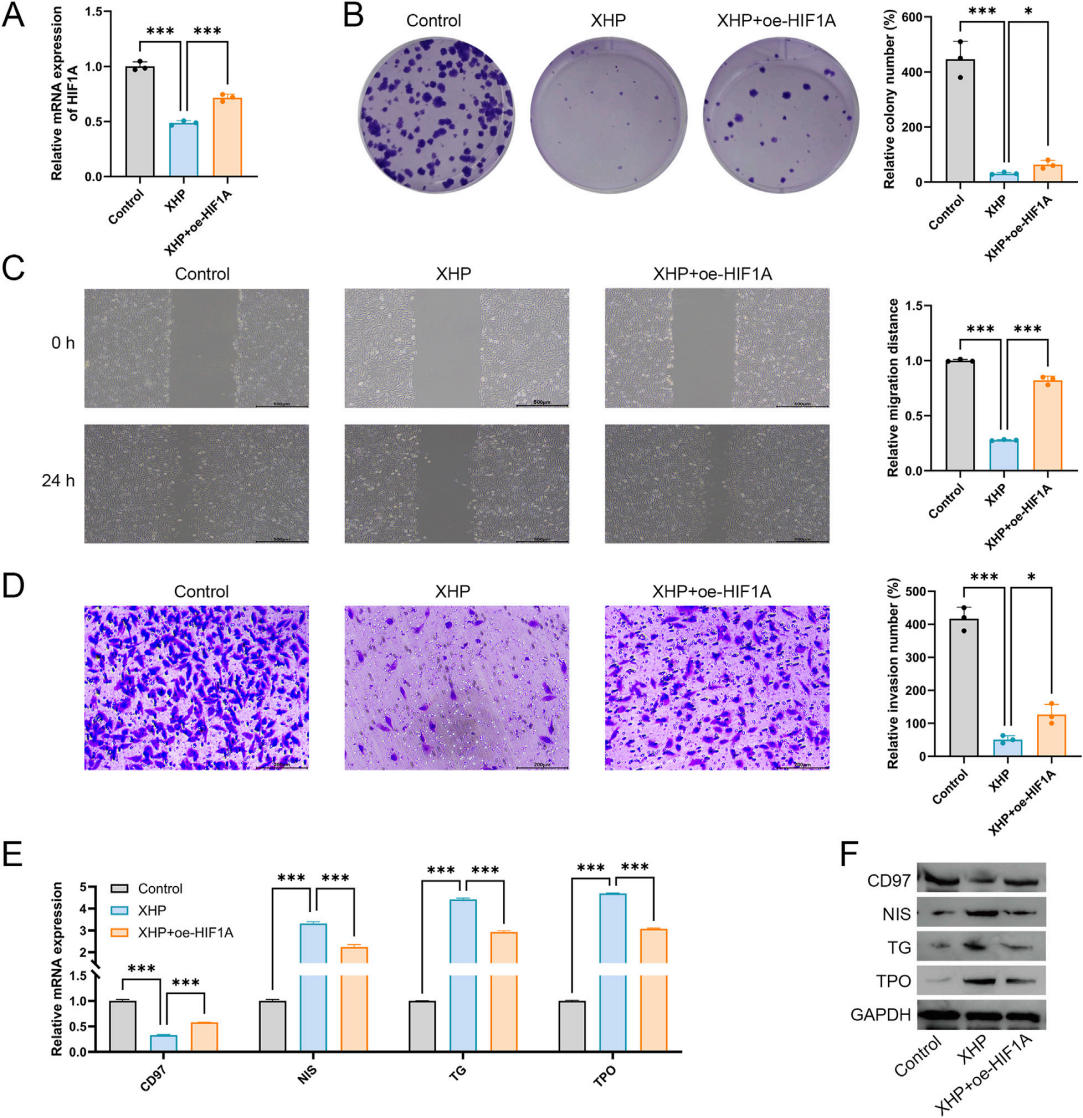
XHP inhibits TPC-1 cell malignant features and promotes differentiation by downregulating HIF1A expression. **(A)** Expression of HIF1A was detected using qRT-PCR. **(B)** Cell proliferation was determined using colony formation analysis. **(C)** Cell migration was determined using wound healing analysis; scale bar = 500 μm. **(D)** Cell invasion was determined using Transwell analysis; scale bar = 200 μm. **(E)** mRNA expression of CD97, NIS, TG, and TPO. **(F)** Protein expression of CD97, NIS, TG, and TPO. TPC-1 cells were treated with 100 μmol/L XHP and transfected with oe-HIF1A. *P < 0.05, ***P < 0.001.

To further investigate whether XHP exerts its therapeutic effect in PTC by targeting HIF1A, HIF1A was overexpressed in XHP-treated TPC-1 cells for feedback validation. As shown in Figure 9A, compared to the XHP group, HIF1A expression was significantly upregulated in the XHP + oe-HIF1A group (P < 0.001), confirming successful overexpression of HIF1A. Consistent with previous results, compared to the control group, XHP significantly suppressed the proliferation, migration, and invasion of TPC-1 cells (P < 0.001, Figures 9B–D). However, HIF1A overexpression reversed these inhibitory effects, promoting the proliferation, migration, and invasion of XHP-treated TPC-1 cells (P < 0.05, Figures 9B–D). Furthermore, HIF1A reversed the differentiation-promoting effect of XHP on TPC-1 cells. HIF1A overexpression significantly increased CD97 expression while suppressing the expression of NIS, TG, and TPO in XHP-treated cells (Figures 9E,F).

### 3.8 The relationship between HIF1A and clinical characteristics of patients

The above analyses have indicated the importance of HIF1A in the development of PTC, and it may be a potential therapeutic target of XiHuang Pill in the treatment of PTC. We further explored the association of HIF1A with the clinical characteristics of patients. The baseline characteristics of patients grouped by HIF1A expression level were presented in Table 4. A total of 501 patients were included in the study, with 305 in the low HIF1A expression group and 196 in the high expression group. There were no significant differences between the two groups in terms of age, gender distribution, racial composition, tumor depth, tumor length, or tumor width. However, the disease duration was significantly longer in the high HIF1A expression group compared to the low expression group (P = 0.002). Notably, there was a significant difference in the distribution of histopathological subtypes (P < 0.001). The high HIF1A expression group had a significantly higher proportion of classic subtype (83.505% vs. 64.765%) and tall cell subtype (12.887% vs. 3.691%), whereas the follicular subtype was more common in the low expression group (31.544% vs. 3.608%). The proportion of patients with advanced-stage disease was significantly higher in the high expression group (41.237% vs. 28.197%, P = 0.006). Additionally, a greater proportion of patients in the high expression group had more than eight lymph nodes involved (43.949% vs. 33.043%, P = 0.030), and the proportion with ≥4 positive lymph nodes was also significantly higher (35.032% vs. 28.509%, P < 0.001). In contrast, a higher proportion of patients in the low expression group had no positive lymph nodes (51.754% vs. 33.121%). The distribution of anatomical tumor locations also differed significantly between the groups (P = 0.019), with bilateral and isthmus involvement being more common in the high expression group. Finally, there were no significant differences between the two groups in terms of personal medical history and primary tumor lesion type.

**TABLE 4:** The baseline characteristics of patients with PTC grouped by HIF1A expression level.

Variables	Subgroup	Low (n = 305)	High (n = 196)	P
age		46 [35, 57]	47 [35, 58]	0.963
tumor depth		1.5 [1.1,2.0]	1.5 [1.0,2.0]	0.572
tumor length		2.5 [1.5,4.0]	2.5 [1.7,4.0]	0.408
tumor width		2.0 [1.4,3.0]	2.0 [1.5,3.0]	0.669
duration		1.0 [1.0,3.0]	2.0 [1.0,4.0]	0.002
gender	male	88 (28.852)	47 (23.980)	0.230
	female	217 (71.148)	149 (76.020)	
race	Hispanic	206 (91.556)	155 (89.080)	0.404
	non-Hispanic	19 (8.444)	19 (10.920)	
histopathological type	classical	193 (64.765)	162 (83.505)	<0.001
	follicular	94 (31.544)	7 (3.608)	
	tall cell	11 (3.691)	25 (12.887)	
clinical stage	early	219 (71.803)	114 (58.763)	
	advanced	86 (28.197)	80 (41.237)	
examined lymph nodes number	≤8	154 (66.957)	88 (56.051)	0.030
	>8	76 (33.043)	69 (43.949)	
positive lymph nodes number	0	118 (51.754)	52 (33.121)	<0.001
	1–3	45 (19.737)	50 (31.847)	
	≥4	65 (28.509)	55 (35.032)	
anatomic site	left lobe	102 (33.663)	73 (38.021)	0.019
	right lobe	144 (47.525)	68 (35.417)	
	bilateral and isthmus	57 (18.812)	51 (26.563)	
personal medical history	normal	161 (63.386)	119 (73.006)	0.093
	nodular hyperplasia	47 (18.504)	19 (11.656)	
	lymphocytic thyroiditis	46 (18.110)	25 (15.337)	
primary neoplasm focus type	unifocal	162 (54.730)	103 (52.821)	0.678
	multifocal	134 (45.270)	92 (47.179)	

The variables showing differences between the 2 groups were selected for exploring their association with the HIF1A expression by univariable linear regression analysis, and the results (Table 5) found that except the anatomic site, the disease duration, histopathological types of PTC, examined number of lymph nodes, and positive lymph nodes number were related to the HIF1A expression (all P < 0.05). Subsequent multivariable linear regression analysis found that histopathological types and positive lymph nodes number were independently related to the HIF1A expression (all P < 0.05). It can be seen that the expression of HIF was also related to many other clinical features and may be closely involved in the development of the disease.

**TABLE 5:** The association of variables with the HIF1A expression by linear regression analyses.

Variables	Univariable		Multivariable	
	OR (95% CI)	P	OR (95% CI)	P
duration	0.024 [0.007,0.041]	0.006	0.010 [-0.011, 0.031]	0.370
histopathological type				
classical	reference		reference	
follicular	−0.582 [-0.716, 0.448]	<0.001	−0.469 [-0.644, −0.294]	<0.001
tall cell	0.211 [0.004, 0.419]	0.046	0.191 [-0.039, 0.420]	0.105
examined lymph nodes number				
≤8	reference		reference	
>8	0.160 [0.025, 0.296]	0.020	0.061 [-0.109, 0.231]	0.481
positive lymph nodes number				
0	reference		reference	
1–3	0.411 [0.248, 0.573]	<0.001	0.210 [0.041, 0.379]	0.015
≥4	0.291 [0.140, 0.442]	<0.001	0.092 [-0.104, 0.288]	0.357
anatomic site				
left lobe	reference		reference	
right lobe	−0.112 [-0.241, 0.018]	0.092	−0.063 [-0.210, 0.083]	0.396
bilateral and isthmus	0.150 [-0.006, 0.305]	0.059	0.106 [-0.060, 0.271]	0.213

## 4 Discussion

In this study, we initially obtained 132 common targets associated with the XHP ingredients and PTC. KEGG enrichment analysis showed that 132 genes were involved in PI3K-AKT, FoxO, and HIF-1 signaling pathways. PI3K-AKT is a common signal pathway that regulates cell growth and proliferation in the development of cancers (Ediriweera et al., 2019), and AKT could affect tumor cell apoptosis through regulating apoptosis-related protein levels (Alzahrani, 2019). Currently, some mRNAs and miRNAs have been identified to be involved in the proliferation, metastasis and apoptosis in THCA, such as IGF, TBK1, TRIP13 and miR-1246 (Lv et al., 2021; Jiang et al., 2023; Li et al., 2022; Yu et al., 2022). Besides, FoxO has recently been identified as an essential factor in the cell cycle and apoptosis (Karger et al., 2009), and several studies claimed that the FoxO transcription level was inhibited by the PI3K-AKT axis (Franz et al., 2016). HIF-1 is a downstream gene of the PI3K-AKT signal pathway, and its expression was upregulated through activating the PI3K-AKT signal pathway (Gao et al., 2020). Several studies identified that HIF-1 played a role in tumor cell proliferation and apoptosis in cancers (Gao et al., 2020; Jin et al., 2022; Jiang et al., 2020).

Based on the 132 targets and ingredients, we constructed a PPI network. We found that the key ingredients were the MOL001063 (28-acetoxy-15α-hydroxymansumbinone) and MOL001027 (myrrhanone A), which all belonged to Mo Yao. Currently, there is no study reporting the function of MOL001063 and MOL001027 in the treatment of cancer. In addition, AKT1 and HIF1A were finally identified as the key oncogenes in the development of PTC. The progression of PTC was largely related to the dedifferentiation of cancer cells with the development of disease. Therefore, we also explored their role in the PTC dedifferentiation, finding that only HIF1A was associated with the differentiation level of patients. Considering its favorable prognostic role, we speculated that HIF1A can promote the dedifferentiation in PTC, thus promoting the disease aggravation. The study showed that HIF-1A was not detectable in normal tissue but was expressed in THCA, and dedifferentiated anaplastic tumor (ATCs) exhibited higher levels (Burrows et al., 2009). Another study also showed that hypoxia score was a significant indicator for dedifferentiation status in THCA, and targeting HIF1A can inhibit aggressive phenotype of dedifferentiated THCA (Ben et al., 2022). The high expression of HIF1A indicates that the tumor environment is in a state of hypoxia. Hypoxia can induce YAP activation, accelerate glycolysis in PTC cells and reduce NIS expression (Hongjun et al., 2023). NIS is a key marker of thyroid cell differentiation, suggesting that hypoxia reduces the differentiation level of PTC cells. These studies strongly supported our current findings that overexpression of HIF1A played a critical role in the dedifferentiation of PTC.

In the process of tumor deterioration, the changes in immune microenvironment are also an essential aspect. We also assessed the immune microenvironment changes associated with HIF1A. The results indicated that HIF1A was negatively related to the abundance of activated NK cells. It has been revealed that NK cells are the main immune effector, which can mediate differentiation of different cancer stem cells through lysis and secretion of interferon γ and tumor necrosis factor α (Anahid et al., 2020). This study pointed out the influence of NK cells on tumor differentiation. Immune infiltration analysis also suggested that the increase of HIF1A in PTC may induce the dedifferentiation of tumor cells and promote the tumor deterioration by inhibiting the activity of NK cells.

Our cellular experiments confirmed that XHP may suppress the malignant characteristics of TPC-1 cells and promote their differentiation by downregulating the expression of HIF1A. Notably, inhibition of HIF1A has been recognized as a promising therapeutic strategy in various types of cancer (Rashid et al., 2021). To date, several HIF1A inhibitors have been identified and evaluated in both preclinical and clinical settings. For example, PX-478 is a well-studied small-molecule inhibitor that suppresses HIF1A synthesis. It has demonstrated antitumor activity in several cancers, including prostate and pancreatic cancers, and has been tested in a Phase I clinical trial (Palayoor et al., 2008). Additionally, liposome-encapsulated echinomycin, a HIF-1α inhibitor, has been shown to eliminate established metastases in triple-negative breast cancer (Bailey et al., 2020). Ban et al. developed the HIF1A inhibitor IDF-11774, which inhibits HSP70 chaperone activity and prevents the accumulation of HIF1A. This compound demonstrated significant anticancer effects in mouse models harboring KRAS, PTEN, or VHL mutations (Ban et al., 2017). Furthermore, Peng et al. identified a marine-derived HIF1A inhibitor, Yardenone 2, which suppresses the proliferation of prostate cancer cells by reducing HIF1A nuclear localization at the protein level (Peng et al., 2024). In studies related to thyroid cancer, 2-methoxyestradiol (2ME2), a promising anticancer agent currently under clinical investigation, was found to enhance the antitumor efficacy of cabozantinib against THCA cells both *in vitro* and *in vivo* (Lin et al., 2016). Beyond direct inhibition of HIF1A, combined therapeutic strategies targeting both the HIF1A pathway and immune modulation have also demonstrated synergistic potential. It has been reported that inhibition of HIF1A can restore NK cell immune activity (Ni et al., 2020). In light of our findings that high HIF1A expression is associated with reduced NK cell activity and tumor dedifferentiation, we propose that targeting HIF1A may serve as a “dual-purpose” strategy: reversing dedifferentiation while restoring immune responsiveness within the tumor microenvironment. Our finding highlights the central role of HIF1A in promoting dedifferentiation and immune evasion in PTC. Further exploration of HIF1A-targeted therapies, particularly in aggressive or dedifferentiated PTC subtypes, may provide new therapeutic opportunities. Our study revealed that XHP showed potential in inhibiting HIF1A. Future research should focus on validating the therapeutic efficacy of XHP in targeting HIF1A to suppress PTC progression in preclinical models and clinical settings.

## 5 Conclusion

This study identified 5 core targets associated with XHP and PTC. The HIF1A was finally identified as the key oncogene in PTC, as its expression in PTC was higher than normal group and higher expression of HIF1A was associated with a poor prognosis of patients. HIFA was further found to correlate with the dedifferentiation of PTC and decreased infiltration of NK cells. Our findings suggested that HIF1A may be a useful target of XHP in the treatment of PTC by inhibiting dedifferentiation.

## Data Availability

The raw data supporting the conclusions of this article will be made available by the authors, without undue reservation.

## Supplementary material

SUPPLEMENTARY FIGURE S1 Expression of HIF1A in normal and PTC cell lines. **(A)** mRNA expression of HIF1A was determined using qRT-PCR. **(B)** Protein expression of HIF1A was determined using Western blotting. ***P < 0.001.
