# Supplementary figures and images for: HIF1A acts as target of XiHuang Pill in the treatment of papillary thyroid cancer by regulating dedifferentiation
Source: Front Chem. 2025 Jun 9;13:1607067. doi: 10.3389/fchem.2025.1607067 (PMC12183632; doi:10.3389/fchem.2025.1607067)

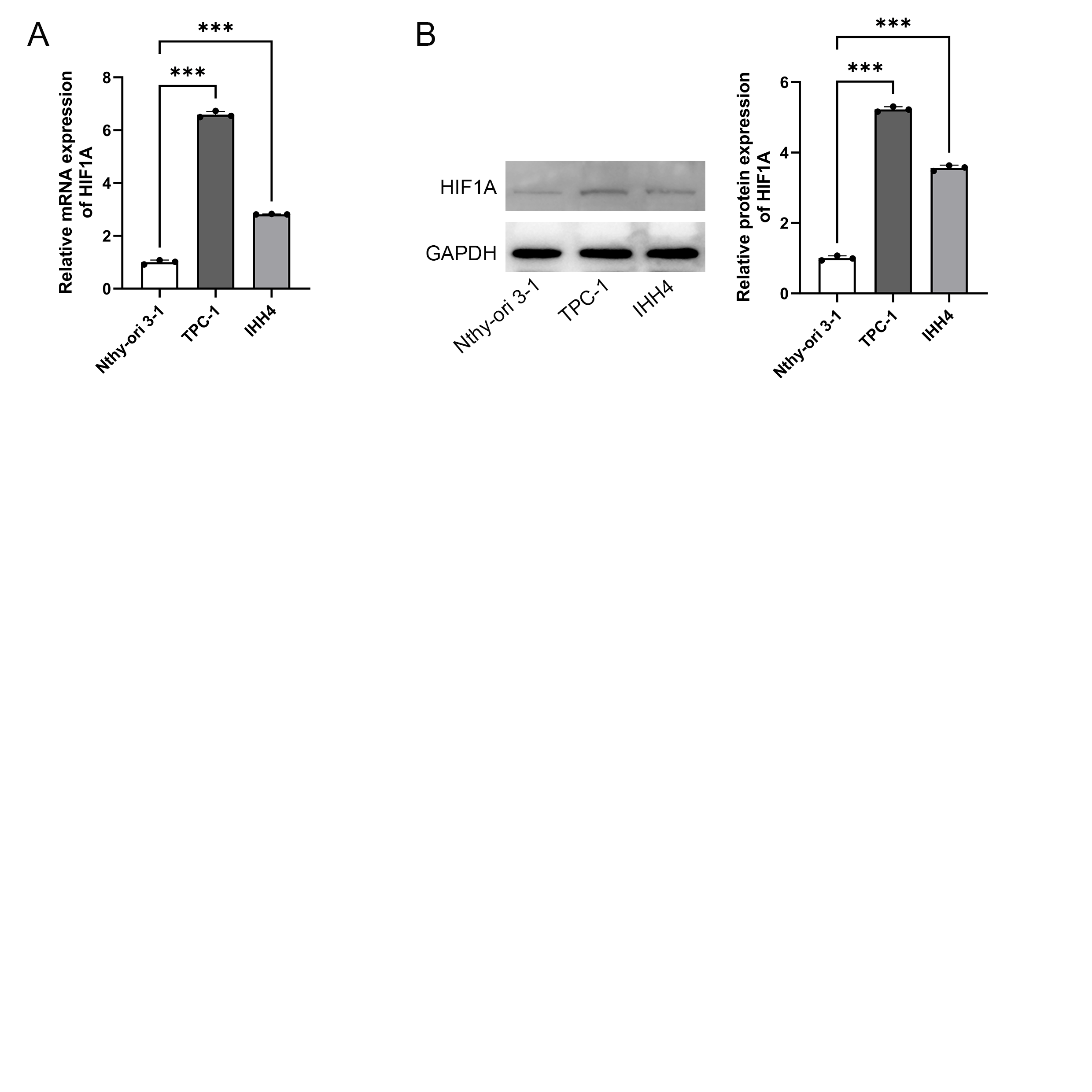

Supplement: Supplementary file 1 [file Image1.tif]
